# Aspirin Modifies Inflammatory Mediators and Metabolomic Profiles and Contributes to the Suppression of Obesity-Associated Breast Cancer Cell Growth

**DOI:** 10.3390/ijms21134652

**Published:** 2020-06-30

**Authors:** Chia-Chien Hsieh, Huai-Hsuan Chiu, Chih-Hsuan Wang, Ching-Hua Kuo

**Affiliations:** 1 Undergraduate and Graduate Programs of Nutrition Science, School of Life Science, National Taiwan Normal University, Taipei 10610, Taiwan; joyce.walawala@gmail.com; 2 School of Pharmacy, College of Medicine, National Taiwan University, Taipei 10051, Taiwan; huaihsuanchiu@ntu.edu.tw (H.-H.C.); kuoch@ntu.edu.tw (C.-H.K.)

**Keywords:** aspirin, adipocytes, breast cancer, inflammation, metabolomics, fatty acid

## Abstract

Breast cancer is the most common cancer among women. Adiposity generally accompanies immune cell infiltration and cytokine secretion, which is ideal for tumor development. Aspirin is a chemopreventive agent against several types of cancer. The aim of this study was to investigate whether aspirin inhibits the growth of 4T1 breast cancer cells by inhibiting the inflammatory response and regulating the metabolomic profile of 3T3-L1 adipocytes. 3T3-L1 adipocyte-conditioned medium (Ad-CM) was used to mimic the obese adipose tissue microenvironment in 4T1 cells. The results revealed that aspirin inhibited macrophage chemoattractant protein (MCP-1), interleukin (IL-6), IL-1β, and plasminogen activator inhibitor (PAI-1) production in 3T3-L1 adipocytes stimulated by tumor necrosis factor-alpha (TNF-α) and lipopolysaccharide (LPS). In the obesity-associated model, Ad-CM significantly promoted 4T1 cell growth and migration, which were attenuated after aspirin treatment. The results of metabolic analyses using Ad-CM showed that amino acid metabolites and oxidative stress were increased in mature 3T3-L1 adipocytes compared to those in fibroblasts. Aspirin treatment modified metabolites involved in suppressing lipogenesis, oxidative stress, and neoplastic formation. In the relative fatty acid quantitation analysis of Ad-CM, aspirin diminished fatty acid contents of C16:1, C18:1, C18:2, C20:4, and C24:1. This study is the first to show that aspirin modifies the metabolomics and fatty acid composition of 3T3-L1 adipocytes and inhibits obesity-associated inflammation that contributes to obesity-related breast cancer cell growth and migration.

## 1. Introduction

Cancer is defined as a group of neoplastic cells that grow uncontrollably and destroy the body’s healthy physiology. Breast cancer is one of the most frequent and ubiquitous cancers throughout every region of the world. In 2018, World Health Organization (WHO) statistics indicated that breast cancer affects 2.1 million women every year and encompasses approximately 15% of all cancer-related mortality amongst women [1]. Epidemiological evidence shows that 30–50% of all cancers are preventable, mainly through changes in lifestyle, environment, and diet [1]. Prevention is considered to be the most effective and economically advantageous strategy in controlling several diseases, especially cancer [1].

Overweight and obesity have become significant impairments to public health, leading to several pathological developments. In 2016, the WHO estimated that over 650 million adults worldwide were overweight or obese, comprising approximately 39% and 13% of the global population, respectively [2]. The main characteristic of adiposity is the hyperaccumulation of fat accompanied by chronic low-grade inflammation. In the adipose tissue microenvironment, several types of immune cells demonstrate increased infiltration into adipose tissue, setting off a series of proinflammatory cytokine secretions, for example, interleukin-6 (IL-6), leptin, and tumor necrosis factor-alpha (TNF-α) [3].

Corroborating evidence exists showing that obesity is associated with cancer development and progression due to chronic inflammation, hormonal imbalances, and metabolic abnormalities [4]. Obesity is recognized as a significant risk for breast cancer development, with obese breast cancer patients shown to have overall lower survival rates and higher risk of recurrence regardless of whether therapeutic interventions are sought [5,6]. Carcinogenesis progression involves the participation of endothelial cells, stromal fibroblasts, tumor cells, and infiltrating immune cells. The recruitment of immune cells, such as macrophages and leukocytes, generates reactive oxygen species (ROS). Along with ROS production, several cytokines contribute to neoplastic transformation, angiogenesis, invasion, and metastasis [4,7].

Metabolomics is a field of science that studies the metabolites generated from blood, body fluids, cells, and tissues through metabolic processes [8]. The number of metabolites in humans is estimated to be in the range of 5000 to 20,000; the majority of these metabolites are not well understood. The pattern of metabolites within subjects was shown to vary due to factors such as environment, heredity, nutrition, sex, and overall physiology. In addition to animal studies, metabolite analyses of cell lines provided better understanding regarding the metabolomics and metabolic pathways of specific cells under different conditions. The contribution of metabolite analyses to preclinical data while minimizing interfering factors has allowed metabolomics analysis of cell lines to be one of the more advanced and innovative preclinical applications for future research [9].

Aspirin (i.e., acetylsalicylic acid) is one of many nonsteroidal anti-inflammatory drugs (NSAIDs) that is widely used for anti-inflammatory, antipyretic, and coronary heart disease protective reasons [10]. Breast cancer tumorigenesis is associated with the inflammatory pathway and overexpression of cyclooxygenase-2 (COX-2). Thus, a possible mechanism of aspirin as a chemopreventive agent in breast cancer may be the inhibition of COX-2, which blocks inflammatory reactions [11]. Recently, aspirin was shown to interfere in the communication between macrophages and 4T1 breast cancer cells, thus suppressing breast cancer cell growth [12]. Accumulated evidence from epidemiological studies, animal experiments, and cell models showed that aspirin exerts chemopreventive properties, especially in breast cancer [13,14]. However, there are still few studies regarding aspirin treatment for breast cancer development induced by obesity.

This work aimed to investigate the mechanism of aspirin chemoprevention in obesity-related breast cancer. The effect of aspirin on the inflammatory response, metabolomic profile, and fatty acid composition in 3T3-L1 adipocytes is tested and the inhibitory effects on the growth and migration of 4T1 breast cancer cells are investigated.

## 2. Results

### 2.1. Aspirin Suppresses Proinflammatory and Angiogenesis-Related Cytokines in 3T3-L1 Adipocytes

**Figure 1:**
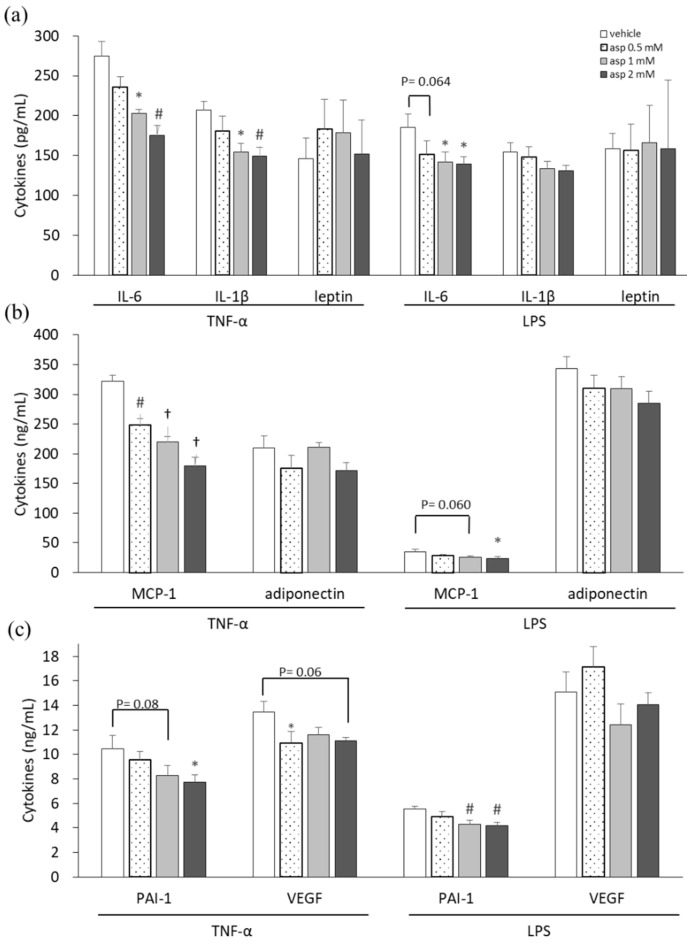
Aspirin inhibited proinflammatory cytokines, adipokines, and angiogenic mediators in 3T3-L1 adipocytes. 3T3-L1 adipocytes were induced with 10 ng/mL tumor necrosis factor-alpha (TNF-α) and 1 μg/mL lipopolysaccharide (LPS) stimulation and treated with various doses of aspirin for 24 h. (**a**) Interleukin-6 (IL-6), IL-1β, and leptin in cell supernatants were analyzed using ELISA. (**b**) Macrophage chemoattractant protein-1 (MCP-1) and adiponectin production were analyzed using ELISA. (**c**) Plasminogen activator inhibitor-1 (PAI-1) and vascular endothelial growth factor (VEGF) production were analyzed using ELISA. Data are presented as mean ± SEM of at least three independent experiments. Statistical analysis was examined by one-way ANOVA and the least significant difference (LSD) post hoc test. Significance of difference is presented as * *p* < 0.05, # *p* < 0.01 or † *p* < 0.001 vs. vehicle group.

In our previous study, a high dose of 5 mM aspirin demonstrated significant cellular toxicity, whereas 2 mM of aspirin did not affect cell numbers but decrease lipid drop accumulation in 3T3-L1 adipocytes [15]. Thus, dosages of 0.5, 1, and 2 mM of aspirin were used in this experiment. Proinflammatory cytokine-, adipokine-, and angiogenesis-related mediators were analyzed in supernatants of 3T3-L1 adipocytes to evaluate the chemopreventive properties of aspirin, as shown in Figure 1. 3T3-L1 cells, in which inflammation was induced by TNF-α and lipopolysaccharide (LPS) stimulation, were treated with various doses of aspirin for 24 h. Subsequently, the culture supernatants were collected and assayed. In the aspirin-treated TNF-α model of 3T3-L1 cells, significant decreases were observed in interleukin-6 (IL-6), IL-1β, macrophage chemoattractant protein-1 (MCP-1), plasminogen activator inhibitor-1 (PAI-1), and vascular endothelial growth factor (VEGF) secretion (*p* < 0.05) compared to the vehicle control. Upon LPS stimulation, cells treated with aspirin revealed decreases in the levels of cytokines IL-6, MCP-1, and PAI-1 in a dose-dependent manner (*p* < 0.05) compared to the vehicle control. In inflammatory models of 3T3-L1 adipocytes, aspirin decreased the secretion of proinflammatory cytokines and angiogenesis mediators. However, aspirin treatment did not affect the secretion of adipokines, such as leptin and adiponectin.

### 2.2. Aspirin Regulates Metabolism in 3T3-L1 Adipocytes

**Figure 2:**
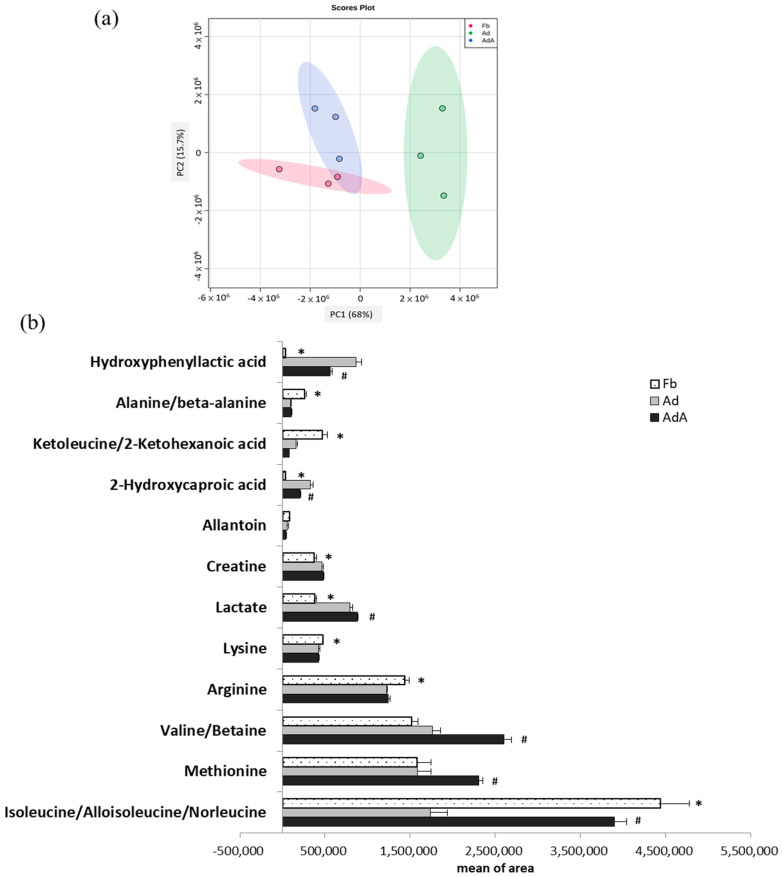
Metabolomics comparison between 3T3-L1 fibroblast- and adipocyte-conditioned media with and without aspirin treatment. Aspirin treatment was added during the differentiation stage along with every medium renewal. (**a**) Principal component analysis (PCA) score plots of fibroblast-conditioned medium (Fb), adipocyte-conditioned medium (Ad), and Ad with aspirin treatment (AdA). (**b**) Metabolites showing significant differences between the three groups were analyzed using one-way ANOVA statistical analysis and the LSD post hoc test. Significant differences are presented as * *p* < 0.05, Fd vs. Ad; # *p* < 0.05, Ad vs. AdA group. Data are presented as mean ± SEM of three independent experiments.

Cultured media were collected for analysis by LC-MS to investigate whether adipocyte differentiation and aspirin intervention caused metabolic changes in 3T3-L1 cells. Metabolite profiling was performed using conditioned media of 3T3-L1 fibroblasts (Fb), mature adipocytes (Ad), and 3T3-L1 fibroblasts treated with 1 mM aspirin during the differentiation process (AdA) (Figure 2). 

In LC-MS analysis, a total 49 metabolites were identified in the Fb, with 45 metabolites identified in the Ad and 59 metabolites identified in the AdA. A total of 28 metabolites were identified in both the Ad and AdA groups and 36 metabolites were identified in both the Ad and AdA groups. The same metabolites that were identified in the Fb, Ad, and AdA groups are shown in Supplementary Table S1. Figure 2a shows the principal component analysis (PCA) score plots of the Fb, Ad, and AdA groups. During fibroblast differentiation into adipocytes, a clear separation between Fb and Ad revealed metabolite profiles that were distinct between these two groups. As the AdA group moved closer to the Fb group, the results indicated that the aspirin treatment reversed the metabolic change in the Ad group. 

One-way ANOVA was further used to identify the significant metabolites between the three groups. Twelve metabolites showed significant differences among the three groups (*p* < 0.05); these metabolites are listed in Figure 2b. Additionally, the least significant difference (LSD) test was used to identify metabolites with significant differences between the two groups; the fold changes and involved pathways of significant metabolites are shown in Table 1. These metabolites are associated with branched-chain amino acid (BCAA) metabolism, alanine metabolism, urea acid/arginine biosynthesis, leucine biosynthesis, phenylalanine metabolism, fatty acid metabolism, and arginine metabolism. Compred to the Fb group, isoleucine (BCAA metabolism), alanine (alanine metabolism), arginine (urea acid/arginine biosynthesis), keto-leucine/2-ketohexanoic acid (leucine biosynthesis) and lysine were decreased by 0.39, 0.37, 0.85, 0.35, and 0.90 times, respectively, in the Ad group (*p* < 0.05). On the other hand, hydroxyphenyllactic acid (phenylalanine metabolism), 2-hydroxycaproic acid(fatty acid metabolism), lactate (energy metabolism), and creatine were increased by 26.8, 9.01, 2.09, and 1.25 times, respectively, in the Ad group (*p* < 0.05).

The metabolites showing significant differences between the AdA group and Ad group were also displayed in Table 1. Isoleucine, valine/betaine, methionine, and lactate were increased by 2.25, 1.48, 1.46, and 1.11 times, respectively, in the AdA group compared to the Ad group (*p* < 0.05), while 2-hydroxycaproic acid and hydroxyphenyllactic acid were decreased by 0.63, and 0.7 times, respectively, compared to the Ad group (*p* < 0.05). Most importantly, this study observed that aspirin treatment reversed the concentration changes of hydroxyphenyllactic acid, 2-hydroxycaproic acid, and isoleucine in the Ad group. The metabolic changes suggested that aspirin might attenuate obesity-related metabolism.

### 2.3. Aspirin Regulates Fatty Acid Composition in 3T3-L1 Adipocytes

**Figure 3:**
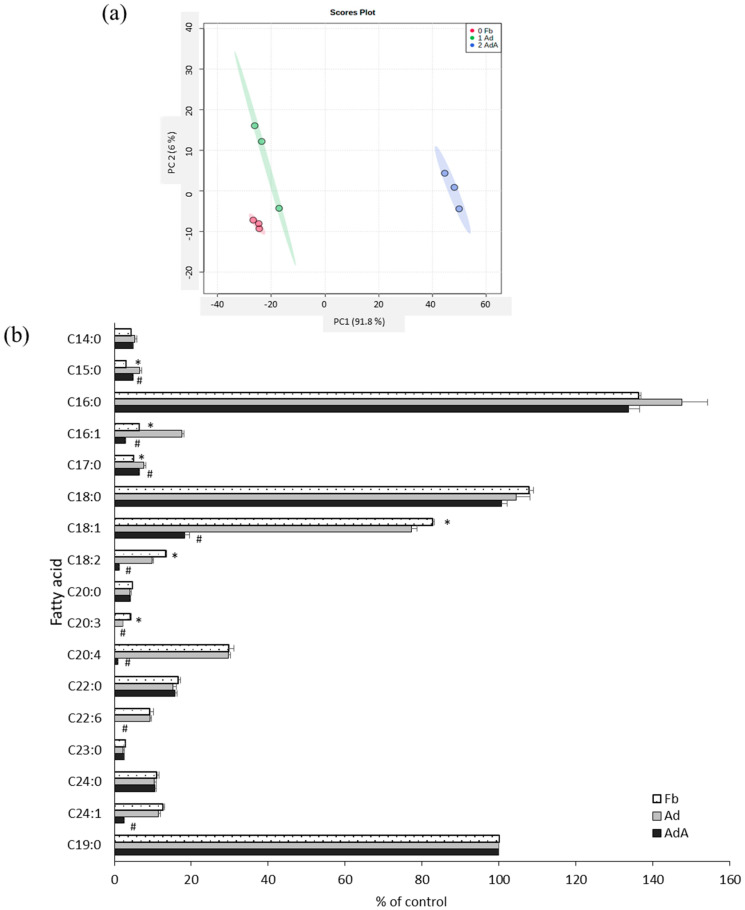
Comparison of fatty acid profiles between 3T3-L1 adipocytes with and without aspirin treatment. The fatty acids in the conditioned media of 3T3-L1 fibroblasts (Fb), adipocytes (Ad), and cells treated with aspirin during differentiation (AdA) were collected and detected by GC-MS. (**a**) The PCA score plots of Fb, Ad, and AdA. (**b**) Fatty acids with significant differences. Data are presented as mean ± SEM of three independent experiments. Statistical analysis was done by one-way ANOVA and the LSD post hoc test. Significant differences are presented as * *p* < 0.05 between Fb vs. Ad and # *p* < 0.05 between Ad vs. AdA.

Fatty acids were profiled using GC-MS to investigate the effect of aspirin on fatty acid metabolism. Figure 3a reveals the PCA score plots of the Fb, Ad, and AdA groups, which displayed clear group sepration according to fatty acid profiles. All data were processed using one-way ANOVA and the LSD test; the fatty acid profiles are listed in Figure 3b. In the Ad group, fatty acids C15:0, C17:0, and C16:1 were increased, but C18:1, C18:2, and C20:3 were significantly decreased compared with the Fb group. C16:1, C18:1, C18:2, C20:4, and C24:1 were significantly decreased (*p* < 0.05) after the addition of aspirin (Figure 3b).

### 2.4. Aspirin Inhibits Ad-CM-Promoted 4T1 Cell Proliferation and Migration

**Figure 4:**
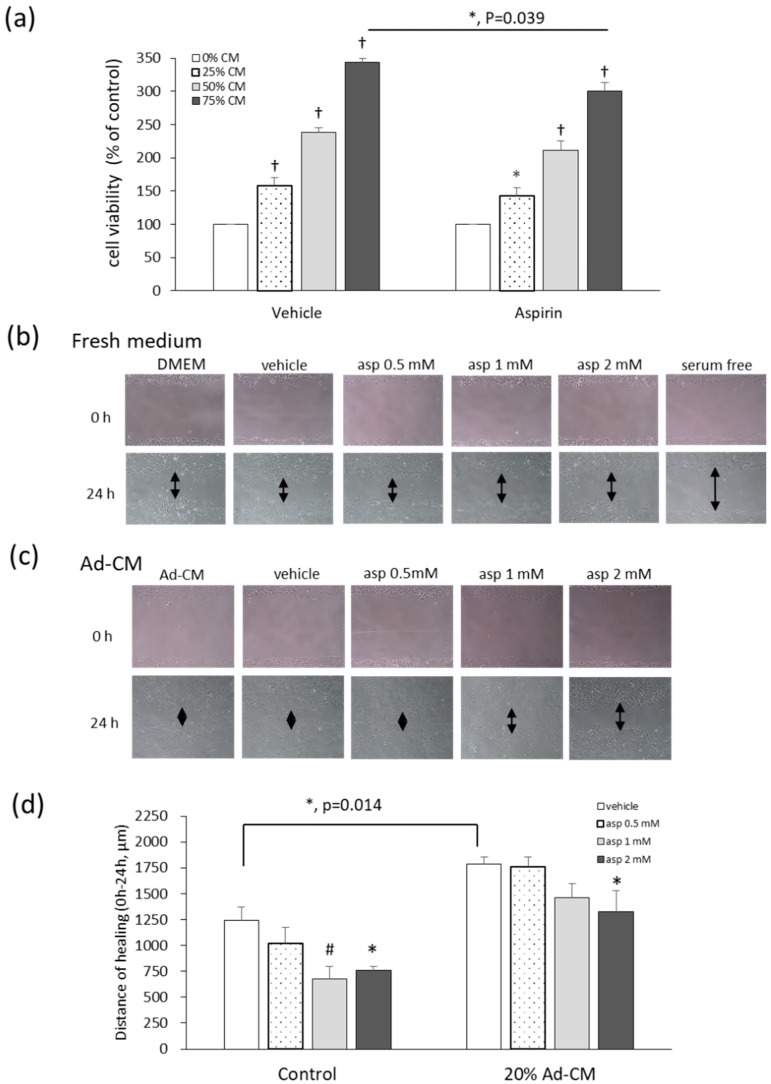
Aspirin suppresses cell growth and migration of 4T1 cells cultured in obese-associated models. (**a**) 4T1 cells were treated with or without 1 mM aspirin, and cultured in different percentages of adipocyte-conditioned medium (Ad-CM) for 72 h. Cell viability was analyzed by the methylthiazole tetrazolium (MTT) test. Migration patterns were observed in the scraped distance of 4T1 cells treated with or without aspirin for 24 h and incubated in (**b**) fresh medium 3% fetal bovine serum (FBS) or (**c**) 20% Ad-CM. The morphology of 4T1 cells was observed microscopically at 100× magnification. (**d**) The distance of healing was quantified by manual measurement under the microscope. The assay was performed with at least three independent experiments. Data are presented as mean ± SEM. Statistical analysis was done by one-way ANOVA and the LSD post hoc test. Significant differences are presented as * *p* < 0.05, # *p* < 0.01, or † *p* < 0.001 vs. vehicle group.

Different levels of adipocyte-conditioned medium (Ad-CM) were added to the 4T1 culture medium, and the methylthiazole tetrazolium (MTT) test was performed in order to evaluate the effects of Ad-CM and aspirin on the viability of 4T1 cells. Cells cultured with an Ad-CM percentage series of 0%, 25%, 50%, and 75%, significantly enhanced the proliferation of 4T1 cells by 59%, 138%, and 243%, respectively, in a dose-dependent manner, compared to cells not treated with CM (Figure 4a). When 4T1 cells were cultured in 75% Ad-CM and treated with 1 mM aspirin, aspirin significantly inhibited the growth of 4T1 cells grown in 75% Ad-CM by 43% (*p* = 0.039) compared to the vehicle group. In our previous study of fresh medium, aspirin also inhibited 4T1 cell proliferation at dosages of 1, 2, and 5 mM by 8%, 20%, and 71%, respectively, in a dose-dependent manner [12,15].

Cell migration was analyzed with a wound-healing assay. The cell monolayer was scratched using a pipette tip and the wound-healing distance was measured. When cells were cultured under serum starvation for 24 h, cell proliferation was absent as a negative control (Figure 4b). After 24 h, treatment with 1 and 2 mM aspirin significantly inhibited healing by 45.5% and 38.6% (*p* = 0.008; *p* = 0.019) after wound scratching compared to the vehicle control (Figure 4b,d). Additionally, in 20% Ad-CM conditioned medium, cell migration significantly increased compared to that observed in fresh medium (*p* = 0.014). Moreover, 2 mM aspirin also inhibited cell migration compared to the vehicle group in Ad-CM conditioned medium (*p* = 0.037) (Figure 4c,d).

## 3. Discussion

Accumulating research and epidemiological studies concluded that obesity increases the risk of breast cancer and therapeutic challenges, while reducing overall prognosis [5,7,16]. In this study, aspirin’s effects on the inflammation and metabolomics of adipocytes and the growth of breast cancer cells were investigated. Aspirin suppressed inflammation-stimulated release of mediators MCP-1, IL-6, IL-1β, and PAI-1 in 3T3-L1 adipocytes. In the obese model, Ad-CM significantly promoted 4T1 cell growth and migration, but aspirin blunted this effect. A possible mechanism leading to these observations was first reported in the metabolic analysis of Ad-CM, wherein aspirin was thought to regulate the metabolomics of lipogenesis, energy metabolism, and oxidative stress, which were shown to be increased in maturate adipocytes. In the fatty acid analysis of Ad-CM, aspirin diminished fatty acid contents of C16:1, C18:1, C18:2, C20:4, and C24:1. In summary, aspirin inhibited obesity-associated inflammation and modified the metabolomics and fatty acid composition of 3T3-L1 adipocytes, contributing to the suppression of obesity-related cell growth and migration of breast cancer. 

Adiposity is generally accompanied by immune cell infiltration and inflammatory cytokine secretion, creating a low-grade inflammatory and angiogenic microenvironment for neoplastic cell development [17,18,19] and contributing to metabolic dysfunction [20]. Additionally, in breast cancer progression, mediators are produced in the tumor microenvironment, such as the proinflammatory cytokines IL-6, MCP-1, and IL-1β, adipokine leptin, the angiogenic mediator PAI-1, and VEGF, which are involved in cell proliferation, migration, and remodeling of the microenvironment [21]. 

The serum levels of MCP-1 and IL-6 in obese individuals are frequently higher than in thin individuals [3]. These proinflammatory cytokines recruit and activate monocytes and trigger an inflammatory response in white adipose tissue [22]. This response was demonstrated to be related to increases in VEGF production in carcinomas [23]. Mice implanted with 4T1 cells demonstrated significantly increased levels of VEGF, TNF-α, and MCP-1 in sera than in mice without tumors [24], indicating that these mediators promote tumorigenesis. In obese subjects with breast cancer, high levels of PAI-1 in serum resulted in increased expression of extracellular matrix proteins, leading to tumor cell attachment and poor prognosis [25]. Therefore, inflammatory and angiogenic indicators are associated with cellular neoplasm and malignancy development, leading to worse prognosis [26], suggesting that breaking the connection between adipocytes and tumor cells might contribute to cancer prevention.

Additionally, aspirin inhibited breast cancer 4T1 cell growth, migration, MCP-1 secretion, and VEGF secretion [15]. Aspirin blunted IL-6 and TNF-α secretion and decreased peroxisome proliferator-activated receptor γ (PPARγ) gene expression in 3T3 fibroblasts cultured with LPS and macrophage-conditioned medium [27]. This suppressive effect of aspirin was demonstrated in both human and animal studies [28]. Here, a model of TNF-α and LPS stimulation was used to mimic inflammation of the adipose tissue environment in order to evaluate the role of aspirin in 3T3 adipocytes, consistent with other findings showing that aspirin significantly decreased proinflammatory and angiogenic mediator secretion in adipocytes, thereby contributing to the inhibition of breast cancer 4T1 cell proliferation and migration [15]. In our previous study, aspirin blocked the communication of RAW264.7 macrophages and 4T1 cells by inhibiting the production of inflammatory and angiogenic mediators, resulting in less breast cancer proliferation [12]. 

Leptin and adiponectin are major adipokines secreted from adipocytes. Besides energy homeostasis, leptin plays a role in immune regulation and the inflammatory response and is involved with metabolic disorders and neoplastic development [29]. In contrast, adiponectin acts inversely and is associated with inflammation and adiposity. The obesity microenvironment demonstrates a higher leptin–adiponectin ratio, with evidence showing that leptin is associated with initiating and progressing cancer development [30]. However, consistently with this study, no research showed that aspirin influenced leptin and adiponectin production.

Metabolite analysis provides an extensive profile, referred to as specific phenotypes and functions, of specific compositions such as obese metabolic disorders [31]. Energy metabolism, fatty acid biosynthesis, BCAA degradation, and phenylalanine metabolism were reported to be dysregulated in abnormal metabolic obesity [32]. During 3T3-L1 differentiation, glucose levels decreased, whereas lactate increased in the culture medium as a result of the disposal of excess glucose within adipocytes [33]. Moreover, obese subjects and diabetes patients present higher concentrations of lactate in plasma compared to healthy individuals [34], indicating that lactate concentration is related to obesity and insulin resistance. Based on previous evidence, the metabolite changes in our study are further discussed herein.

Our results indicated that compared to the Fb group, isoleucine decreased in the Ad group; this result was consistent with previous reports [35,36]. Isoleucine, one of three BCAAs, is mainly used by skeletal muscle and adipocytes to increase mitochondrial content, resulting in an enhanced oxidative reaction [37]. The metabolites involved in BCAA metabolism during adipocyte differentiation are consumed, participate in the citric acid cycle, and increase lipid production. Green et al. observed that differentiated adipocytes increased BCAA catabolism during adipocyte proliferation [35]. Additionally, it was observed in diabetic (db/db) mice supplemented with BCAAs that macrophage infiltration and gene expression of inflammatory hormones in white adipose tissue decreased compared to the control group [38]. In this study, increased lactate and decreased BCAAs were observed in 3T3-L1 adipocytes. The decreased levels of BCAAs in the Ad group were found to be reversed by aspirin, revealing that aspirin modifies the metabolism of 3T3-L1 adipocytes and decreased oxidative stress.

Methionine is a raw material for cell growth and protein synthesis, and participates in single carbon metabolism and methylation. Breast cancer MCF-7 cells treated with methionine were shown to inhibit cell proliferation by reducing p53 expression [39]. This study found that methionine increased after aspirin treatment. In addition, hydroxyphenyllactic acid is derived from phenylalanine, which is involved in adipocyte differentiation. Phenylalanine derivatives are associated with various diseases, such as metabolic dysfunction. Phenylalanine levels were significantly different between metabolically healthy individuals and metabolically abnormal obese patients [32]. Obese individuals were shown to possess higher levels of serum phenylalanine. In an early study, mice administered p-hydroxyphenyllactic acid showed an increase in malignant tumors, such as adenomas, leukemias, hepatomas, and vascular tumors [40]. Hydroxyphenyllactic acid was also shown to increase ROS in mitochondria, causing oxidative stress [41]. Moreover, higher levels of 3-(4-hydroxyphenyl)lactate were linked to energy metabolism dysfunction, insulin resistance, and oxidative stress in cohort studies [40]. This study found that increased hydroxyphenyllactic acid in 3T3-L1 adipocytes was reversed by aspirin, suggested that aspirin potentially attenuates obesity-related metabolism.

According to our study, fatty acid metabolism changed after aspirin treatment; increases in 2-hydroxycaproic acid, a medium-chain fatty acid involved in lipid metabolism and energy storage [42], in 3T3-L1 adipocytes were reversed after aspirin treatment, suggesting that aspirin suppressed 2-hydroxycaproic acid, thereby decreasing lipid synthesis and storage. Also, higher saturated fat levels were shown to promote inflammatory reactions, neoplastic processes, cancer development, and poorer outcomes [43]. Index ratios of C16:1/C16:0 and C18:1/C18:0 were shown to be positively correlated with body mass index (BMI) and negatively correlated with insulin sensitivity [44]. In this study, the ratio of C16:1/C16:0 in the aspirin group was reduced by 0.18-fold compared with the Ad group, and C18:1/C18:0 was significantly reduced by 0.25-fold, suggesting that aspirin treatment indirectly benefits insulin sensitivity.

Arachidonic acid (AA, C20:4) is synthesized from the essential fatty acid linoleic acid (C18:2) in humans. An AA derivative was determined to trigger and regulate inflammation involved in several diseases [43]. In particular, eicosanoids, which are biologically active metabolites of AA, increased the levels of two series of prostaglandins and four series of leukotrienes [45,46], and demonstrated an association with cancer progression [43]. Aspirin is a well-known COX-2 inhibitor which inhibits downstream inflammatory mediators [47]. Interestingly, the reductions in C18:2 and C20:4 fatty acid contents by aspirin treatment were previously proposed to contribute to interrupting communication between adipocytes and cancer cells, thereby providing a reason for the growth reduction of 4T1 cells observed in this study.

Adiposity is linked with metabolic dysfunction and many chronic disturbances. During this progression, excess fatty acids and nutrients are produced, which can be utilized by surrounding cells, especially tumor cells [3,4,5]. A previous study on 3T3-L1 adipocytes co-cultured with MCF-7 and MDA-MB-231 cells revealed increases in breast cancer cell growth and migration, mainly due to the cancer cells effectively absorbing fatty acids for energy [48]. This was consistent with our results, where Ad-CM promoted 4T1 cell growth and migration. Mazid and coworkers reported that aspirin treatment during adipocyte maturation significantly decreased triacylglycerol and fat accumulation [49], thereby inhibiting the differentiation and lipid accumulation of 3T3-L1 adipocytes [15]. This possible mechanism demonstrated that aspirin inhibits p53 pathways, further inactivating the pentose phosphate pathway and consequently suppressing adipose accumulation [50]. Aspirin treatment in 4T1 cells may decrease free fatty acid metabolism, block lipid formation, and affect the composition of fatty acids. Further research is needed to clarify the relevant molecules and mechanisms involved. Corroborating evidence proposed that aspirin exerts potential chemopreventive and therapeutic properties in many cancers. Aspirin is further thought to play roles in inflammatory-related pathways, hormones, platelets, PI3 kinase, AMP-activated protein kinase, and histone formation [28,47]. Currently, aspirin is entering phase III clinical randomized trials regarding improved survival in breast cancer [51]. In observational studies, routine aspirin was shown to prevent distant metastases and progression of several cancers, reduce distant recurrence rates, and decrease mortality due to breast cancer [52,53]. Moreover, meta-analyses also showed that aspirin has anti-breast cancer properties [14,54]. Based on these pieces of evidence, aspirin likely affects obesity-related disorders by protecting against platelet formation and coronary heart disease [10], inhibiting inflammatory progression [11,12,28,47], suppressing lipogenesis [15,49,50] and neoplastic formation [13,14,51,52,53,54]. However, the unfavorable side effects, particularly its increased likelihood of causing ulceration and bleeding in the gastrointestinal tract, should be considered before the clinical use of aspirin. Therefore, the optimal dosage, duration, format, and cancer type to protect against should be further investigated in order to obtain a standard for precision therapy and to improve efficacy.

## 4. Materials and Methods 

### 4.1. Cell Culture and Reagents

**Figure 5:**
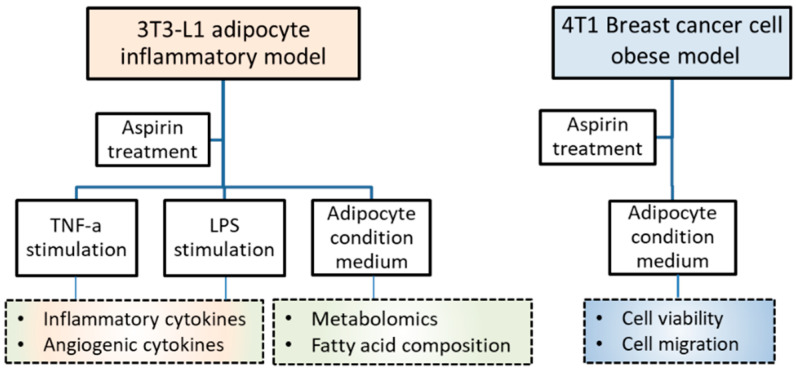
Experimental design and basal conditions.

Mouse fibroblasts 3T3-L1 were purchased from the Bioresource Collection and Research Center (BCRC; Hsinchu, Taiwan) and murine breast cancer 4T1 cells were purchased from the American Type Culture Collection (ATCC; Manassas, VA, USA). Both types of cells were cultured in Dulbecco’s modified Eagle’s medium (DMEM) (Caisson, Smithfield, UT, USA) containing 10% bovine serum (BS) (Gibco, Grand Island, NY, USA) or fetal bovine serum (FBS) (Genedirex, Las Vegas, NV, USA), respectively, with 1% penicillin/streptomycin/amphotericin B (Caisson). Incubation was performed at 37 °C with 5% CO_2_ humidification. Acetylsalicylic acid (Aspirin) (Sigma, St. Louis, MO, USA) was dissolved in dimethyl sulfoxide (DMSO) (Sigma) as a stock and stored at −20 °C. The experimental design included 3T3-L1 adipocyte inflammatory, breast cancer, and obesity-related models, as shown in Figure 5.

### 4.2. T3-L1 Preadipocytes Differentiate into Adipocytes

3T3-L1 fibroblasts were seeded in 10% FBS/DMEM until full confluence for 2 days. Cells then underwent induced differentiation (day 0) by culturing in 10% FBS/DMEM containing 25 mM glucose, 10 μg/mL insulin (Sigma), 0.5 mM 3-isobutyl-1-methylxanthine (Sigma) and 0.2 μM dexamethasone (Sigma) for 4 d. Then, the medium was changed to 10% FBS/DMEM containing 25 mM glucose; 10 μg/mL insulin and media were replaced every 3 days for 13 days, where fibroblasts were considered as mature adipocytes for further analysis.

### 4.3. Cytokines, Adipokines, and Angiogenic Mediators in 3T3-L1 Adipocytes

Mature 3T3-L1 adipocytes were treated with various doses of aspirin and stimulated by 2.5 ng/mL TNF-α (PeproTech, Rocky Hill, NJ, USA) and 1 mg/mL lipopolysaccharide (LPS) (Sigma) in DMEM containing 1% BS for 24 h. Cell culture supernatants were collected and cytokine levels were analyzed by enzyme-linked immunosorbent assay (ELISA). The analytic protocol of cytokines included IL-6, IL-1β, macrophage chemoattractant protein-1 (MCP-1), leptin, adiponectin, plasminogen activator inhibitor-1 (PAI)-1 (R&D, Minneapolis, MN, USA), and vascular endothelial growth factor (VEGF) (PeproTech, Rocky Hill, NJ, USA), according to the manufacturer’s instructions. 

### 4.4. Obesity-Associated Model Set-Up 

The 3T3-L1 adipocyte-conditioned medium (Ad-CM) was prepared to mimic the obese environment in vitro. 3T3-L1 cells induced differentiation at day 12, and the medium was replaced with DMEM for 24 h. Then, the adipocyte-conditioned medium (Ad-CM) was collected for in vitro subsequent studies. In the other model, 4T1 cells were treated 200 ng/mL leptin (Recombinant Mouse leptin protein, R&D) for 24 h to mimic the obese environment in vitro, as referred to in our previous studies [15,18].

### 4.5. Metabolite Analysis of 3T3-L1 Adipocyte-Conditioned Medium 

The conditioned medium from 3T3 fibroblasts, adipocytes, and cells treated with aspirin during differentiation were collected to investigate the small molecular metabolites of adipocytes. Briefly, 3T3-L1 fibroblasts were placed in a 6-well plate at 1.5 × 10^5^/well, and the medium was changed to 10% FBS/DMEM during the differentiation stage. The culture supernatant was collected on the first day and named the fibroblast (Fb) group. The medium was changed on days 0, 4, 7, and 10 during the differentiation process, where 1 mM aspirin was subsequently added. The culture supernatant was collected on the 13th day, named the adipocyte (Ad) group, and cells treated with aspirin comprised the (AdA) group. The supernatant was collected and stored at −20 °C for analysis of small molecule metabolites and relative quantitative analysis of fatty acids. 

### 4.6. Metabolite Profiling by Liquid Chromatography–Time of Flight Mass Spectrometry 

The samples of conditioned media were extracted by a methanol: H_2_O mixture (4:5, *v/v*). Then, small molecule metabolites were analyzed by the Metabolomics Core Laboratory, Centers of Genomic and Precision Medicine (National Taiwan University, Taipei, Taiwan) using liquid chromatography–mass spectrometry. Briefly, samples were injected into UHPLC–MS/MS, an Agilent 1290-UHPLC coupled with an Agilent 6540 quadrupole time-of-flight (QTOF) mass system (Agilent, Santa Clara, CA), for mass detection. The analytical column was an Acquity High strength silica (HSS) T3 column (2.1 × 100 mm, 1.8 μm, Waters, Milford, MA). The mobile phase included water/0.1% formic acid and acetonitrile/0.1% formic acid with a 300 μL min^−1^ flow rate. The gradient consisted of 0–1.5 min, 2% B; 1.5–9 min, 2–50% B; 9–14 min, 50–95% B; 14–17 min, 95% B. The column re-equilibration time was 3 min. The injection volume was 2 µL. A jet stream electrospray ionizing source was used for sample ionization. The following parameters were used throughout the study: gas temperature, 325 °C; gas flow, 8 L min^−1^; nebulizer, 40 psi; sheath gas temperature, 325 °C; sheath gas flow, 10 L min^−1^; capillary voltage, 4 kV for positive and 3.5 kV for negative; and fragmentor, 120 V. The mass scan range was m/z = 50–1700. Peak identification was conducted by matching m/z and retention time to an established inhouse database, i.e., the National Taiwan University MetaCore Metabolomics Chemical Standard Library containing 348 metabolites. A total 49 metabolites were identified in the Fb, 45 metabolites were identified in the Ad, and 59 metabolites were identified in the AdA sample extracts.

### 4.7. Fatty Acid Analysis by GC-MS

For the gas chromatography–mass spectrometry (GC-MS) analysis of C14 to C26 fatty acids, collected samples were extracted by the Folch method and derivatized using acetyl chloride. The C19:0 was used as the internal standard. All analyses were performed in spitless mode using an Agilent 7890a gas chromatograph connected to an Agilent 5975C Series mass. selective detector (MSD) (Agilent, Santa Clara, CA). The chromatographic columns were 30 m DB-5 MS + DG capillary columns (5% phenyl, 95% dimethylpolysiloxane), with an internal diameter of 250 μm (Agilent, Santa Clara, CA). The injection volume was 1 μL. The oven temperature began at 60 °C for 1 min and was then increased to 160 °C at a rate of 30 °C min^−1^, followed by a further increase to 260 °C at a rate of 2 °C min^−1^, then to 300 °C at 5 °C min^−1^, which was maintained for 1 min. The equilibration time between injections was 2 min. The electron impact ionization was 70 eV. The MS source and MS quadrupole were maintained at 230 °C and 150 °C, respectively. The masses of the analysis were acquired in full scan mode with a mass range of 30–650 m/z.

### 4.8. T1 Cell Viability Assay

Cells at a concentration of 1.5 × 10^3^/well in 96-well plates (Becton Dickinson) were utilized to investigate the effect of Ad-CM on the viability of 4T1 cells. Cells were cultured in various concentrations of Ad-CM replacement in fresh 1% FBS/DMEM medium without or with 1 mM aspirin. The FBS concentration was adjusted to be the same in each group. After 72 h of treatment, cells were replaced by 0.5 mg/mL methylthiazole tetrazolium (MTT) solution (Sigma) for 3 h at 37 °C. Then, the solution was moved, and DMSO was added to solubilize the MTT formazan crystals for 15 min on the shaker. The absorbance of 540 nm was measured using a microplate spectrophotometer (BioTek). The data were presented as percentages of the control. Cell viability was calculated using the Equation (1).

(1) $$\frac{\mathrm{ODsample}-\mathrm{ODblank}}{\mathrm{ODcontrol}-\mathrm{ODblank}}\;\times \;100=\mathrm{Cell}\;\mathrm{viability}\;\%\;\mathrm{of}\;\mathrm{control}$$

### 4.9. T1 Cell Migration Assay 

A wound healing assay was used to evaluate the migration of 4T1 cells. 4T1 cells were plated at a density of 8.6 × 10^4^ cells/well in 24-well plates in 3% FBS/DMEM until cells reached 90% confluence. A 10 mL pipette tip scratched the cell layer, and the media were replaced by the presence or absence of aspirin under three conditions, namely, fresh medium, 200 ng/mL leptin medium, or 20% Ad-CM medium. Cell images were taken at 100× magnification using a microscope-connected camera (WS500, Whited, Taoyuan, Taiwan) at 0 and 24 h. 

### 4.10. Statistical Analysis

All data were collected from at least three independent experiments, presented as the mean ± standard error of the mean (SEM). Student’s *t*-test tested significant differences between the two groups. The differences between the groups were tested using one-way analysis of variance (ANOVA), followed by the least significant difference (LSD) post hoc test using Statistical Product and Service Solutions (SPSS) version 19 (IBM Corp., Armonk, NY, USA,). A *p*-value of less than 0.05 was considered to be statistically significant.

## 5. Conclusions

**Figure 6:**
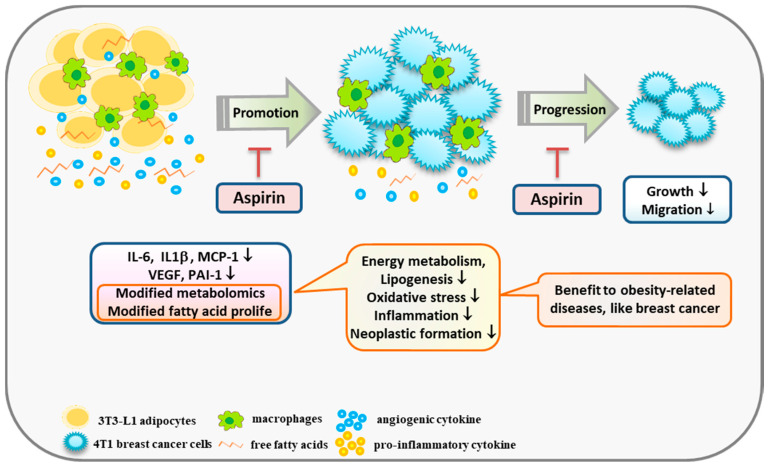
A scheme displaying a possible mechanism by which aspirin modifies obesity-related mediators and metabolomics profiles, resulting in suppression of the growth of breast cancer cells. Aspirin inhibits IL-6, IL-1β, MCP-1, and PAI-1 production by 3T3-L1 adipocytes under TNF-α and LPS stimulation. In addition, aspirin modifies metabolites involved in suppressing lipogenesis, oxidative stress, and neoplastic formation, and diminished fatty acid contents of C16:1, C18:1, C18:2, C20:4, and C24:1 in adipocyte-conditioned medium. In summary, aspirin suppressed the growth and migration of 4T1 breast cancer cells by regulating obesity-related mediators.

Aspirin truncated the effect of obesity effect 4T1 cell growth and migration by suppressing the inflammatory and angiogenic mediators of adipocytes, as well as cutting off communication between adipocytes and breast cancer cells. Furthermore, to our knowledge, the present study is the first to observe that aspirin treatment during adipocyte differentiation modifies metabolites involved in amino acid metabolism and fatty acid derivatives in media, suggesting that aspirin has a role in suppressing lipogenesis, regulating energy metabolism, and lowering oxidative stress and neoplastic formation (Figure 6). Moreover, due to aspirin’s supportive role in chemoprevention, obese subjects who regularly use aspirin prophylactically for cardiovascular disease may also benefit from prevention of other obesity-related diseases.

## Figures and Tables

**Table 1:** Significant metabolites and involved pathways in 3T3-L1 cells.

Metabolites	Ad/Fb ^1^			AdA/Ad ^1^		Major Metabolic Pathway ^3^
	ANOVA ^2^	Fold ^2^	LSD ^2^	Fold	LSD	
Hydroxyphenyllactic acid	<0.001	26.8	<0.001	0.7	0.002	phenylalanine metabolism
2-Hydroxycaproic acid	<0.001	9.01	<0.001	0.63	0.003	fatty acid metabolism
Lactate	<0.001	2.09	<0.001	1.11	0.030	energy metabolism metabolite
Creatine	0.012	1.25	0.010	1.02	0.664	participates in enzymatic reactions
Isoleucine/Alloisoleucine /Norleucine	<0.001	0.39	0.002	2.25	0.001	valine, leucine, and isoleucine metabolism
Ketoleucine/2-Ketohexanoic acid	0.001	0.35	0.001	0.48	0.169	leucine biosynthesis
Valine/Betaine	<0.001	1.16	0.091	1.48	<0.001	valine, leucine, and isoleucine metabolism
Arginine	0.007	0.85	0.004	1.01	0.755	urea cycle/arginine biosynthesis
Methionine	0.013	1.00	1.000	1.46	0.008	cysteine and methionine metabolism
Alanine/beta-alanine	<0.001	0.37	<0.001	1.09	0.586	beta-alanine metabolism
Lysine	0.033	0.90	0.021	1.00	0.993	biotin metabolism, carnitine synthesis
Allantoin	0.043	0.71	0.103	0.70	0.213	product of the oxidation of uric acid

^1.^ Conditioned media of 3T3-L1 fibroblasts (Fb), adipocytes (Ad), and cells treated with aspirin (AdA) during differentiation were collected and analyzed by LC-MS. ^2.^ Fold change was calculated as the mean of peak intensity of metabolite in every group. The Ad compared to the Fb as Ad/Fb, the AdA compared to the Ad as AdA/Ad. Statistical analysis was done by one-way analysis of variance (ANOVA) and least significant difference (LSD) post hoc test. ^3.^ The major metabolic pathways were identified using the Kyoto Encyclopedia of Genes and Genomes (KEGG) database and the Human Metabolome Database (HMDB).

## Supplementary Materials

Supplementary materials can be found at https://www.mdpi.com/1422-0067/21/13/4652/s1. Supplementary Table S1: Metabolites present in conditioned media of 3T3-L1 fibroblasts, adipocytes, and cells treated with aspirin. The data used to support the findings of this study are included within the article and are available from the corresponding author upon request.

## Abbreviations

AA	Arachidonic acid
Ad	Adipocyte
AdA	Adipocyte treated with aspirin
Ad-CM	Adipocyte-conditioned medium
BCAA	Branched-chain amino acid
COX-2	Cyclooxygenase-2
ELISA	Enzyme-linked immunosorbent assay
Fb	Fibroblast
GC-MS	Gas chromatography-mass spectrometry
IL	Interleukin
LC-MS	Liquid chromatography-mass spectrometry
LPS	Lipopolysaccharide
MCP-1	Macrophage chemoattractant protein-1
MTT	Methylthiazole tetrazolium
PCA	Principal component analysis
PAI-1	Plasminogen activator inhibitor-1
ROS	Reactive oxygen species
TNF-α	Tumor necrosis factor-alpha
VEGF	Vascular endothelial growth factor
WHO	World Health Organization
