# Supplementary material for: Aspirin Modifies Inflammatory Mediators and Metabolomic Profiles and Contributes to the Suppression of Obesity-Associated Breast Cancer Cell Growth
Source: Int J Mol Sci. 2020 Jun 30;21(13):4652. doi: 10.3390/ijms21134652 (PMC7369784; doi:10.3390/ijms21134652)
Supplement: Supplementary file 1 [file ijms-21-04652-s001.pdf]

Supplementary Table 1. Metabolites present in conditioned media of 3T3-L1 fibroblasts, adipocytes, and cells treated with aspirin [1]

Metabolite	ANOVA[2] P value	Fb Mean ± SEM	Ad Mean ± SEM	AdA Mean ± SEM
Pyroglutamic acid	0.937	9115725 ± 258818	9078389 ± 815404	9368075 ± 633775
Phenylalanine	0.174	6953934 ± 356486	6626024 ± 361236	7559456 ± 159134
Pyrrolidonecarboxylic acid	0.604	4487392 ± 161750	4330132 ± 467996	4908888 ± 493332
Tyrosine	0.063	4847854 ± 433049	3977029 ± 363792	5358302 ± 60832
Isoleucine/Alloisoleucine/Norleucine	<0.001	4444666 ± 335676	1734727 ± 201955	3898020 ± 149212
Methionine	0.013	1584779 ± 159622	1584779 ± 159622	2309072 ± 43830
Betaine/Valine	<0.001	1517188 ± 77605	1763222 ± 97403	2607530 ± 83125
6-Hydroxydopamine	0.155	1493825 ± 162490	1293370 ± 113782	1662136 ± 16749
Arginine	0.007	1441605 ± 51587	1222433 ± 8528	1238475 ± 29893
Tryptophan	0.212	1000424 ± 111351	845252 ± 73760	1060176 ± 18280
Ureidopropionic acid	0.206	492466 ± 40613	426456 ± 26469	504367 ± 13669
Lysine	0.033	472775 ± 10958	425988 ± 14264	425857 ± 4519
Lactate	<0.001	379949 ± 20342	793222 ± 28062	877914 ± 12084
Creatine	0.012	372332 ± 25251	465833 ± 15688	477439 ± 9185
Glycerophosphocholine	0.062	164066 ± 14684	99816 ± 9113	113928 ± 21241
Cytosine	0.158	118314 ± 20407	104856 ± 7339	78910 ± 1620
Histidine	0.096	114490 ± 3517	113263 ± 9242	90031 ± 7940
Folic Acid	0.548	111118 ± 16498	107341 ± 10391	124950 ± 2520
Allantoin	0.043	81033 ± 6689	57595 ± 12474	40583 ± 4819
2-Hydroxycaproic acid	<0.001	36755 ± 1156	331154 ± 30104	207695 ± 7422
Ketoleucine/2-Ketohexanoic acid	0.001	465306 ± 63822	162463 ± 15430	78625 ± 3766
Alanine/beta-alanine	<0.001	262799 ± 17329	96844 ± 4483	105481 ± 4111
Hydroxyphenyllactic acid	<0.001	32140 ± 1939	861690 ± 66131	564638 ± 21000

1. The conditioned media of 3T3-L1 fibroblasts (Fb), adipocytes (Ad), and cells treated with aspirin (AdA) during the differentiation were collected and analyzed by LC-MS.

2. Data was present as the mean of peak intensity of metabolite in every group. Statistical analysis was done by one-way analysis of variance (ANOVA).
